# Solidification of Gelatine Hydrogels by Using a Cryoplatform and Its Validation through CFD Approaches

**DOI:** 10.3390/gels8060368

**Published:** 2022-06-10

**Authors:** Yasir Beeran Pottathara, Miha Jordan, Timi Gomboc, Blaž Kamenik, Boštjan Vihar, Vanja Kokol, Matej Zadravec

**Affiliations:** 1 Faculty of Mechanical Engineering, University of Maribor, Smetanova Ulica 17, SI-2000 Maribor, Slovenia; yasir.potta@um.si (Y.B.P.); miha.jordan@student.um.si (M.J.); timi.gomboc@um.si (T.G.); blaz.kamenik@um.si (B.K.); vanja.kokol@um.si (V.K.); 2 IRNAS—Institute for Development of Advanced Applied Systems, Ltd., Limbuška Cesta 76b, SI-2000 Maribor, Slovenia; bostjan@irnas.eu

**Keywords:** gelatine, hydrogel, cryoprinting, CFD simulation, solidification modelling

## Abstract

In this work, we developed a numerical approach based on an experimental platform to determine the working conditions on a cryoplatform and to predict and evaluate the cryogenic printing of hydrogels. Although hydrogels have good biocompatibility, their material properties make it difficult to print them with high precision and shape fidelity. To overcome these problems, a cryogenic cooling platform was introduced to accelerate the physical stabilisation of each deposited layer during the printing process. By precisely controlling solidification (crystallisation), each printed material can withstand its own weight to maintain shape fidelity, and the porosity of the scaffolds can also be controlled more selectively. The thermophysical properties of gelatine hydrogels were investigated to gain a better understanding of the phase change upon freezing. The corresponding material properties and experimental observations of gelatine solidification served as the basis for developing a computational fluid model (CFD) to mimic the solidification of gelatine hydrogels using a cryoplatform at different process conditions and extruder speeds. The goal was to develop a tool simple enough to predict acceptable process conditions for printing gelatine hydrogels using a cryoplatform.

## 1. Introduction

Three-dimensional (3D) bioprinting technology has gained extensive attention because of the peculiarities of a high level of reproducibility and great control on pore distribution and pore size, with good interconnectivity [1]. Three-dimensional bioprinting offers the formation of artificial tissue constructs from the deposited hydrogels with appropriate cell functionalities and mechanical properties, with accurate spatial control in a layer-by-layer pattern to be beneficial for replicating its in vivo counterparts [2,3,4]. For the printing of hydrogels, an extrusion-based 3D printing technique is utilised to improve the resolution and to sustain the mechanical stability of the printed scaffolds throughout a multilayer printing process. The physical stabilisation of each hydrogel deposit can, therefore, withstand its own weight to retain shape fidelity and improve its resolution from a more precise and controlled porous structure to the final scaffold. As proposed in our previous work, a lower-temperature cooling platform would help to stabilise hydrogel deposits if the temperature of the cooling platform is lower than the hydrogel’s crystallisation temperature [5].

Unidirectional freezing and freeze-drying techniques have been employed to fabricate scaffolds with interconnected macro- and micro-scale pores [6,7]. The columnlike architecture of unidirectionally frozen scaffolds is employed in cartilage applications and tailored easily to match that of the native cartilage by varying the material concentration and freezing rates [6]. The cryoplatform for the deposition of printed scaffolds could solve the current limitations, including the size limitation in the case of soft hydrogels, slower printing process, and the storage and preservation of printed scaffolds, and thus enable a more robust scaffold with manageable size requirements through faster printing. It was reported that freezing can enable shape fidelity and thus improve the mechanical properties of the printed scaffolds [8]. A cryoprinting platform can freeze the deposited hydrogels effectively, and enables larger scaffold structures without deterioration, as well as improves their resolution during the printing. The storage and preservation also could be much more effective because of the distinct freezing of each layer of the scaffold rather than as a whole structure. Some of the previous reports of cryoprinting of biological materials utilised the incorporation of cryofluid to create ice-controlled scaffolds [9,10].

The thermophysical parameters of the hydrogel play an important role in the development of a cryoplatform in terms of heat transfer through contact from the cooling platform to the sample, and, later, heat conduction through the sample. If the thermophysical parameters are not correct, the temperature distribution over the sample will be different and the prediction of solidification will be wrong. Gelatine has been chosen as the hydrogel material to perform the validation of the cryoplatform, as it is used widely as a glazing agent in the food [11], pharmaceutical [12], and biological [13] industries, with outstanding biocompatibility and biodegradability. Gelatine derives as a mixture of peptides and proteins, attained through partial hydrolysis of collagen extracted from the skin and bones of animals. Typically, gelatine dissolves easily in warm water (>40 °C), and can form a homogeneous gel in water for about 1–50 wt% concentrations [14]. In this study, we took a sample where the concentration of gel in water was 10%. For a gelatine solution of 10% by weight, it has already been shown that this non-Newtonian, shear-thinning fluid remains in the linear viscoelastic region longer than when we take solutions with a lower concentration [14,15,16]. Different concentrations of gel in water also strongly affect the storage modulus, which is an important parameter in printing [5] and increases with higher concentrations of gel in water. However, gelatine shows low printability to obtain a macroscale or complex pore architecture [17] due to its sol–gel transition properties. During sol–gel transition, gelatine undergoes a phase transition from a liquid to a solid state through its gel point. Gelatine was reported as a thermally reversible gel with water, having a gel state ≈below 25 °C and a solution state ≈above 35 °C [18]. This means that if a gelatine solution is cooled down rapidly below the sol–gel transition temperature, the protein coils convert to triple helices, and the solution transforms to a three-dimensional (3D) gel form having strength and elasticity [19]. On the other hand, when the temperature of the gel is raised above the sol–gel transition temperature, approximately 30 °C, a reverse transition might take place of the helix to coil [20]. In practice, gelatine hydrogels are usually prepared from embedded cells. In this work, the research focused on pure gelatine hydrogels. Therefore, the survival rate of the cells, which is highly dependent on the properties of the bioink such as the pH of the bioink, the specifications of the nozzle, and the pressure applied to the cells, was not the subject of the research. In addition to the above parameters affecting cell survival, the temperature of the cryoplatform would also need to be controlled to prevent a decrease in cell viability.

In the present study, the main objective was to develop a sustainable numerical model of gelatine solidification based on computational fluid dynamic (CFD), which is validated, and can later on also be used to simulate gelatine behaviour (solidification) in accordance with the thermal conditions on the cryoplatform. For that purpose, the material (thermal and flow) properties were measured, and, later on, the freezing kinetics of gelatine solutions were also performed to validate the developed CFD solidification model of the gelatine solution. Finally, a numerical model was built to study the printing parameters (extruder speed) at the given freezing parameters on the cryoprinting platform during 3D gelatine printing and to determine the effects on the temperature distribution during solidification, which may also lead to product deformation due to the change in storage modulus, which is highly dependent on temperature.

## 2. Results and Discussion

### 2.1. Thermophysical Properties of a Gelatine Solution

**Figure 1:**
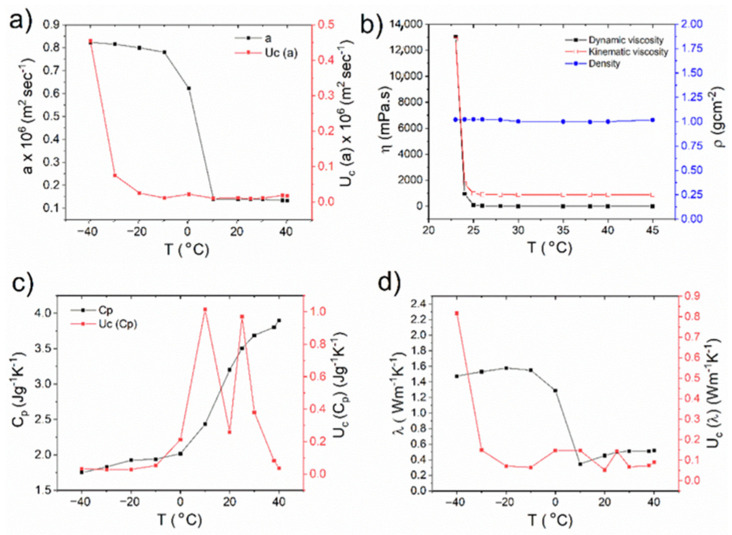
The thermophysical properties of the Gel-10% solution: (**a**) Thermal diffusion density; (**b**) viscosity; (**c**) specific heat; (**d**) calculated thermal conductivity with their corresponding measurement uncertainties ($U_{c}$).

In the CFD simulation of gelatine solidification, cooling is of great importance, and depends largely on the heat flow from the gelatine sample to the cooling plate. To solve this properly, the gelatine material’s properties are important for the design and CFD analysis of the cryoplatform. The material properties of the gelatine solution needed to describe the physical temperature situation are the thermal conductivity *λ*, the specific heat *c_p_*, and the heat of solidification (the latent heat of fusion) $L_{v}$, which is released during the phase transform from the liquid to the solid state of the gelatine solution. Additional to the heat transfer of the gelatine solution is the material, which also flows at some conditions, especially at larger temperatures when it is not solidified. Flowability is a function of density *ρ* and dynamic viscosity *μ*, and the kinematic viscosity *ν* can be derived from them. It should be noted that these properties depend on the local temperature and constitution of the gelatine sample. The variations in the measured thermal and flow properties for a 10 wt% of gelatine solution (termed as Gel-10%) with its temperature are shown in Figure 1, and the corresponding values are listed in Table 1, except for the latent heat of fusion $L_{v}$, which must be calibrated in the CFD simulation according to the performed experiment of gelatine solution solidification. For all the measured temperature material properties, the measuring uncertainties are also given on the secondary axis of the graphs ($U_{c}$) in Figure 1.

Thermal diffusivity is the significant material property for heat transfer, and is crucial in the transient heat diffusion into the material. For a homogeneous isotropic material, the thermal diffusivity is defined as $a=\lambda /\left(\rho \cdot c_{p}\right)$, where λ, ρ, and $c_{p}$ are the thermal conductivity, density, and specific heat capacity of the material, respectively. For Gel-10%, at higher temperatures from 10 °C to 40 °C, the temperature dependence of the thermal diffusivity (Figure 1a) values was almost constant, 0.14 × 10^6^ m^2^/s. At the temperatures lower than 10 °C the thermal diffusivity started to increase with respect to freezing, and attained a maximum value of 0.78 × 10^6^ m^2^/s at −40 °C. The density appears to be constant for the entire measured temperature regime, whereas the dynamic and kinematic viscosities (Figure 1b) started to increase from 25 °C upon cooling. These results are in agreement that the gelation of gelatine happened below the room temperature of ~23 °C. Since the thermal diffusivity provides a material’s response with respect to a change in temperature, gelation for Gel-10% happened totally below ~10 °C, and the solution froze completely upon further cooling to −40 °C. The results also prove that the temperature on the top of the proposed cooling platform must be −20 °C to study the rapid freezing of the material to be deposited.

As shown in Figure 1c, the specific heat capacity, $c_{p}$, of Gel-10% decreased from 3.9 J/g·K upon cooling and reached 1.9 J/g·K at a temperature of −20 °C, and showed a minor decrease upon further freezing. From Figure 1d, the calculated thermal conductivities of Gel-10% solution increased with the freezing, and reached the maximum value of 1.5784 W/m·K at −20 °C and decreased slightly afterwards. At room temperature conditions (20 °C), the thermal conductivity of unfrozen gelatine (0.45 W/m·K) is lower than that of pure water (0.6 W/m·K). The thermal conductivity of a material depends not only on the inherent thermal conductivities and volume fractions of the components, but also on the structure of the material or the spatial distribution of the components [21]. For example, in terms of carbohydrates and proteins, measuring inherent thermal conductivities could be difficult because of their heterogeneous nature. The difference in thermal conductivity values of unfrozen and frozen gelatine could be attributed to the difference in intrinsic values being affected by the state and amount of water bound to the gelatine [22].

### 2.2. Experiment on Gelatine Solution Solidification

**Figure 2:**
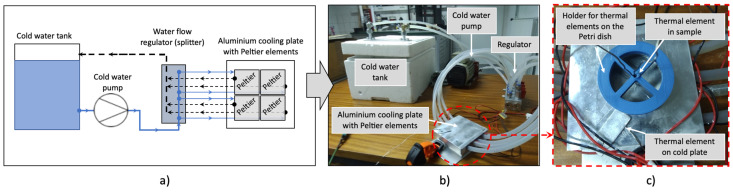
(**a**) Schematic representation of the experimental setup and (**b**) the corresponding digital image of the setup, as well as (**c**) the cryoplatform with the 3D-printed Petri dish and thermocouple holder.

The schematic diagram of the experimental setup for the solidification (cooling) experiments can be found in Figure 2. The gelatine solution solidification experiment is performed to obtain the transient behaviour of the temperature profiles within the gelatine sample to determine the latent heat of fusion $L_{v}$, which is later calibrated in the CFD simulation that mimics the gelatine solution solidification experiment. During the experimental analysis, 12 experiments were performed to see if the experiment was reproducible, and to determine later the average of the measured values, which serve as input and validation values for the numerical modelling. Six temperatures were measured at different locations during the experiment. Thermocouples were used to measure the temperature in the sample at two locations (at the bottom and 2 mm from the bottom), the room temperature, the temperature of the cold plate, and the temperature of the cooling water of the Peltier elements (at the inlet and outlet). The experimental setup consisted of eight Peltier elements, paired in a square arrangement to form four double-layer Peltier pairs. Above the Peltier pairs was the aluminium cryoplatform plate. Below each of the Peltier pairs was a copper heat exchanger, through which the cooling water flowed. The water-cooling system was used to keep the hot side of the Peltier as low as possible, to achieve the lowest temperature on the cold side of the Peltier. An aluminium (Al) Petri dish was placed at the top of the aluminium cryoplatform plate.

**Figure 3:**
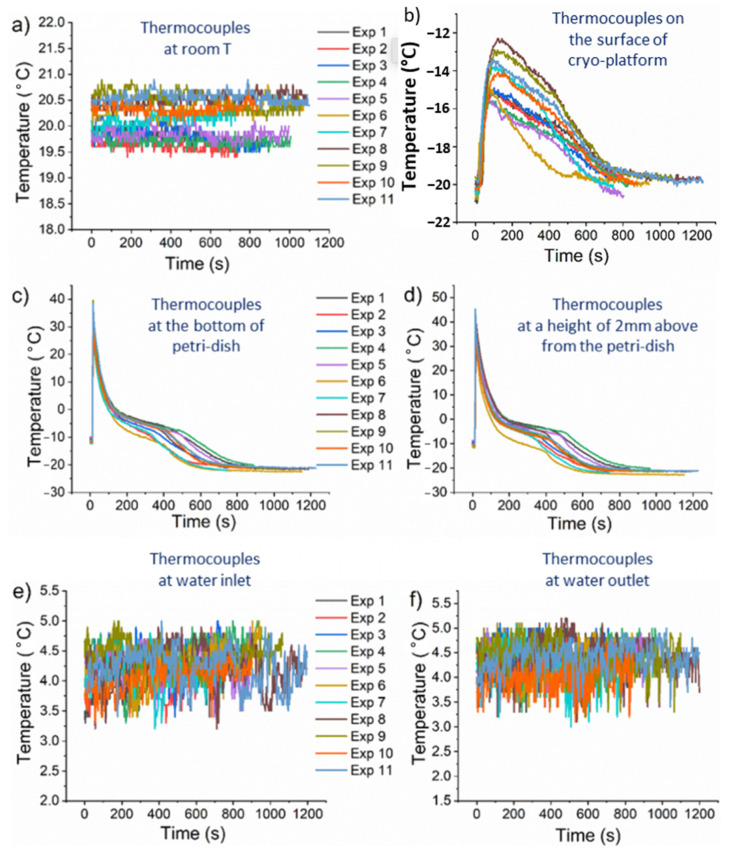
Experimental results of the cooling on the cryoplatform with the Gel-10% solution (different line colors represents 12 reproducible experiments. The cooling kinetics of (**a**) thermocouples at room temperature; (**b**) thermocouples on the surface of the cryoplatform; (**c**) thermocouples at the bottom of the Petri dish; (**d**) thermocouples at a height of 2 mm above the Petri dish; (**e**) thermocouples at the water inlet (water from the chamber); (**f**) thermocouples at the water outlet (water to the chamber).

Once the cryo-platform reached the desired temperature of about −20 °C, the 10% gel solution was poured into the Al-Petri dish on the top of the cryoplatform at a temperature of 40 °C (the gelatine solution was preheated so that it was in liquid form), and the cooling kinetics were recorded with the corresponding thermocouples. The results of the cooling experiments with the designed cryoplatform and the Gel-10% solution are shown in Figure 3. Figure 3a shows the room temperature throughout the measurement period, which ranged from 19.5 °C to 20.7 °C for all twelve experiments. The lowest temperatures in the system were on the cryoplatform. The temperature variations on the cryoplatform during all twelve experiments are shown in Figure 3b. When the temperature on the cryo-platform reached a temperature below −20 °C, the gel solution was added, and the temperature probe positioned on the cryoplatform also registered this, and a sudden but relatively slow increase in the temperature was observed on the cryoplatform from ~−20 °C to ~−12 °C. After the cooling, the power of the Peltier elements was sufficient to compensate for the rapid temperature rise during the addition of the hot gel solution, so the temperature on the cryoplatform started to return to its previous state, and reached the lower stage cooling temperatures again after 800 to 1000 s, when the gel solution was completely frozen. The solidification (freezing) of the gel solution is illustrated by the temperature measurements at the bottom (Figure 3c) and 2 mm above the bottom (Figure 3d) of the Petri dish filled with the gel solution. As mentioned earlier, the temperature at the bottom and 2 mm above the bottom of the Petri dish increased rapidly to a maximum of 40 °C after the addition of the hot gel solution, which corresponds to the filling of the precooled Petri dish with the warm gelatine solution. After filling the gelatine solution, the temperature drops to a lower value. In both positions, the gel temperature reached ~−20 °C in 800 s. In Figure 3e,f, we also monitored the inlet and outlet temperatures of the cooling water used to cool the hot side of the Peltier elements. From the inlet and outlet temperatures of the cooling water, we can see that the inlet temperature was slightly lower than the outlet temperature, and that the temperature variations between the different experiments are in the range of ±1 °C throughout the measurement period. The performed experimental measurements of the temperatures (twelve measurements) were then averaged in time to use these time average values to evaluate the numerical CFD model and calibrate the latent heat of fusion $L_{v}$.

### 2.3. CFD Simulation for Calibration of the Gelatine Material Properties

The cooling performance of the plate on which the biomaterial is printed must be considered in the development phase of the bioprinting platform. Proper and fast solidification is important to obtain the correct structure and shape of the printed model. If the printed gelatine layers solidify slowly, the stability of the underlying layers could lead to mechanical problems of the printed structure. On the other hand, with each new printed layer, we introduce additional heat through the warm liquid gelatine layer on the top of the existing structure, which is the height-limiting factor for heat transfer from the sample to the cooling plate with the notch of the sample. During solidification, heat is extracted from the biomaterial, which must be removed efficiently using a cooling system. In this section, we describe the workflow of a numerical model for cooling composition in 3D printing of biomaterials, and compare the results with the measurement results. Using this CFD model, we calibrated the gelatine latent heat of fusion and thus have a robust and validated modelling tool for observing heat transfer at different process conditions in the bioprinting application (speed of adding a new gelatine layer).

**Figure 4:**
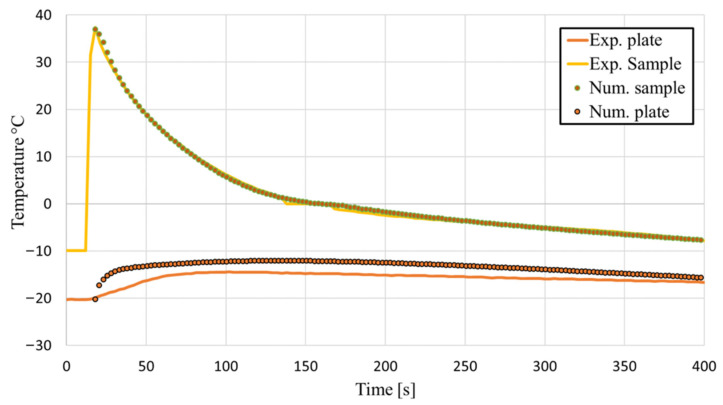
Comparison of the numerical results with the experimental results of the sample temperature 2 mm above the bottom of the sample and the temperature of the plate.

The numerical results were compared in Figure 4 with the experimental freezing kinetics described in Section 2.2. The numerical simulation starts at the moment when the biomaterial is poured into the Petri dish. The initial temperature of the biomaterial is set to 38 °C and the temperature of the plate is set to −20 °C. Transient freezing kinetics were modelled.

As we can observe on Figure 4, the numerically determined temperatures in the middle of the Petri dish, 2 mm above the bottom, agree well with the measured temperatures. In the beginning, the temperature decreased rapidly, due to the fact that the temperature difference between the solid plate and the bio sample was the highest. From about 150 to 170 s, the temperature was approximately constant and then decreased gradually. When we compare the measured temperature of the plate with the numerical results, we see that the numerical results predicted higher temperatures than the experiment. For later use of the final simulation model, the sample temperatures are important, considering that the heat transfer in the gelatine sample is a simulation focus, and therefore, some deviation of plate temperature between the experiment and the simulation can be neglected.

### 2.4. 3D Printing Process Parameter Evaluation

#### Simulation of 3D Printing with a Dynamic Mesh

Once the material properties, the boundary conditions for Peltier cooling, and the solidification model were determined, virtual experiments were performed for three different printing speeds of 500, 750, and 1000 mm/min, where the test gelatine was 10 × 10 mm in size and had a maximum height of 2 mm. The considered printing speeds of 500, 750, and 1000 mm/min (extruder speed) corresponded to volume flow rates of 0.44, 0.66, and 0.88 mm^3^/s of the extruded gelatine material. Since a dynamic mesh is used to simulate the material addition, the initial height of the considered sample corresponds to the extruder diameter, which is 0.26 mm (the initial state is a full layer). In order to observe the heat transfer through the growing gelatine, a simplification was made by adding the entire printing layer at once for all samples distributed over the cooling plate, where the size of the extruder nozzle corresponds to the layer height of 0.26 mm. Such simplification in printing modelling is more conservative in terms of heat transfer, since the larger amount of warm gelatine solution is added at once, and the cold plate has to take over all the heating and solidification energy. If the simulation results show that solidification is too slow under certain process conditions and the added gelatine has not had enough time to cool before the next layer is applied, then, in the real case, one is on the safe side as far as the solidification problem is concerned.

The simplified 3D printing simulation described earlier is performed in four major steps: (a) the material is distributed evenly over the entire cooling surface in the form of fifteen 10 × 10 mm square gelatine samples, (b) a uniform heat flux is applied to the entire aluminium cooling surface, (c) a heat transfer simulation is performed that includes convective heat losses to the environment from the cooling plate and gelatine samples, and (d) the next layer of material is applied to all samples simultaneously, with an additional heat flux source in the new layer mimicking the warm gelatine layer from the extruder. The heat flux resulting from the addition of gelatine material is calculated as follows (Equation (1)):

(1) $$\dot{Q}=\dot{m}c_{p}T=\dot{V}\rho c_{p}\;T=vA\rho c_{p}\;T$$

where T is the temperature of the extruded material equal to 38 °C, $c_{p}$ is the heat capacity of the extruded material, and $v=\dot{\frac{V}{A}}$ is the displacement velocity calculated from the volumetric flows and the sample cross-section. The heat flux defined for the upper region of the sample is equal to:

(2) $$q=\frac{\dot{Q}}{A}=v\rho c_{p}T$$

In the simulations, it was assumed that 15 samples are printed simultaneously, due to the fact that in the case of printing, each sample is positioned at a different location relative to the cooling plate, and this is also one of the impact factors for solidification of each individual sample. Simulation variants which were observed in the numerical experiment are at the different printing speeds of the extruder shown in Table 2.

**Figure 5:**
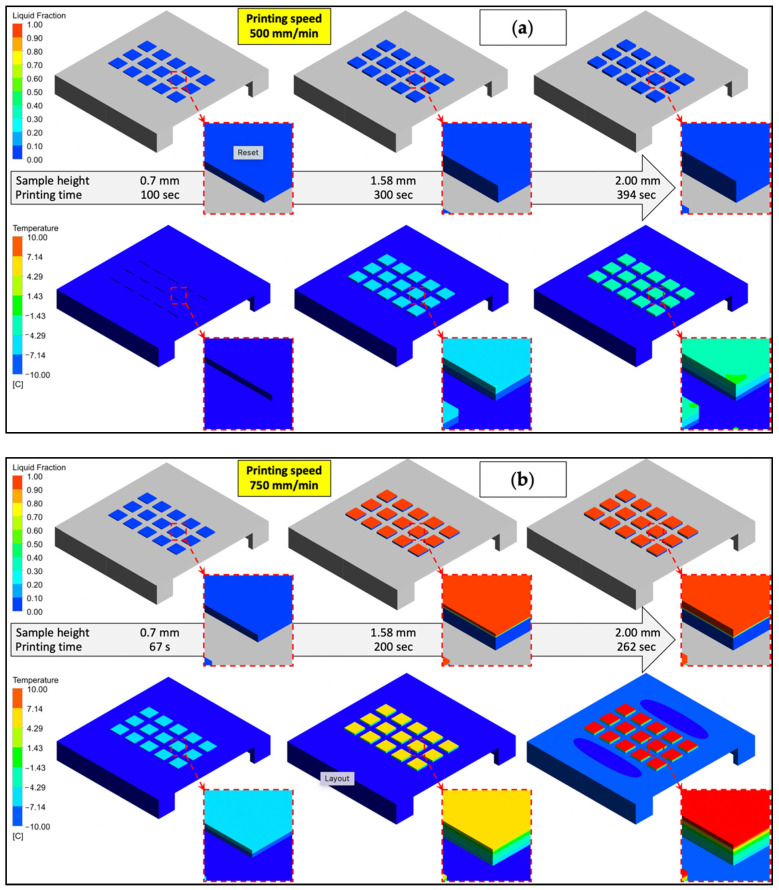

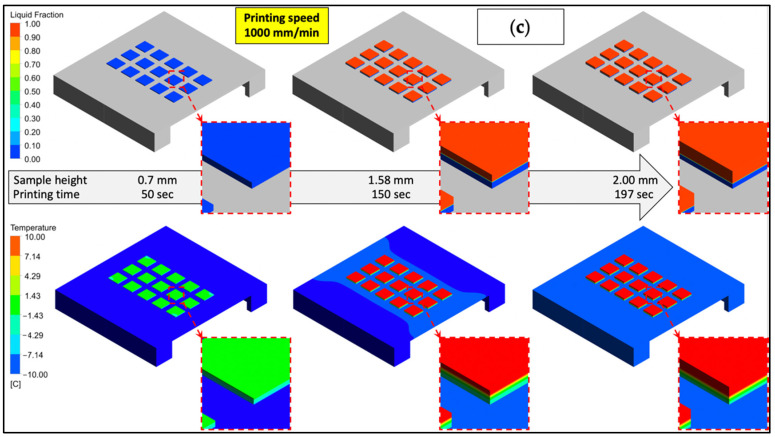
CFD results of liquid fraction and temperature on the sample during different printing speeds for three different printing speeds: (**a**) 500 mm/min, (**b**) 750 mm/min, and (**c**) 1000 mm/min. In all simulation cases the liquid fraction and temperature changed with the printing time of the sample (sample size was 10 × 10 mm and maximum sample height was 2 mm).

As expected, at the lower printing speed (500 mm/min), the bottom lying layers were solidified faster than at the higher printing speed (1000 mm/min) before a subsequent layer was deposited, as shown in Figure 5. This can be seen clearly in the temperature distribution of the simulation figures, as the maximum temperature was higher for the faster printed scaffold (Figure 5c) than for the slower printed scaffold (Figure 5a). At a lower printing speed, each printed layer has sufficient time to cool before a new filament is applied. At each printing speed, with time evolution, the thickness of the gel sample increased, and, therefore, heat conduction increased from the top of the sample where the gelatine solution was added to the bottom of the sample where the cooling was performed, and this can be seen in the temperature contour plots at the top of the sample, which was, in the cases of middle and high printing speeds (Figure 5b,c), above 10 °C.

**Figure 6:**
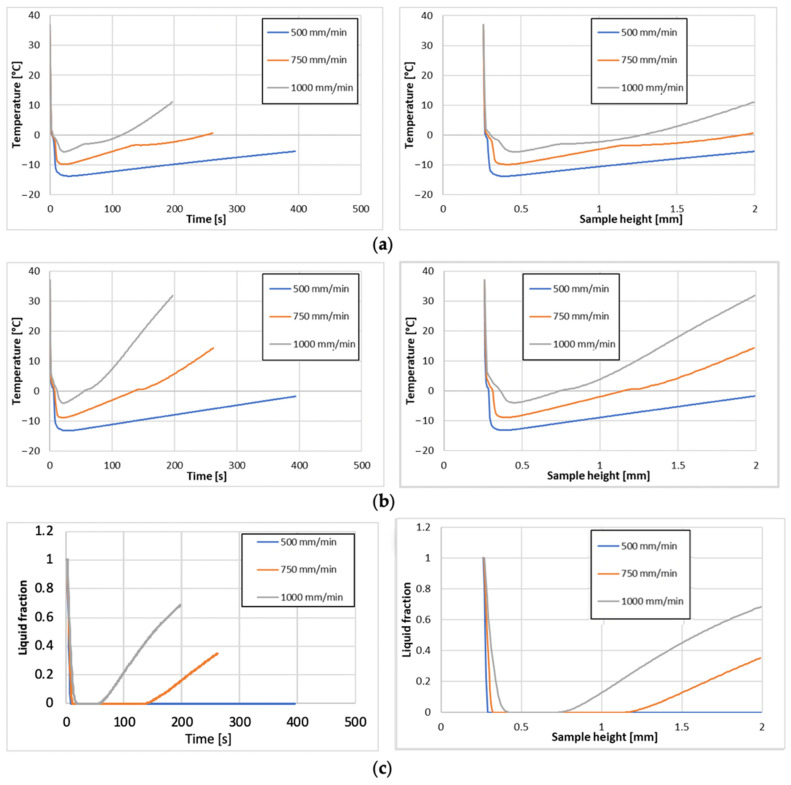
Average temperature (**a**), the average temperature at the top surface (**b**), and an average liquid fraction (**c**) of the bioprinted sample during the printing time (left graphs), or printing sample height (right graphs).

In Figure 6 the liquid fraction and the average temperature at the top of the sample are shown in diagrams to investigate the variation of the freezing process at different printing speeds as a function of printing time and sample height. From the average sample temperatures, we can see (Figure 6a) that at low (500 mm/min) and medium (750 mm/min) printing speeds, we did not overcome the average volume temperature of 0 °C in the printed sample until the end of the final height. This was not the case when we increased the printing speed to the highest observed value (1000 mm/min), where we reached the average volume temperature of 0 °C after only 115 s, or when the height of the printed sample was 1.25 mm (62.5% of the total sample height). The same trends, observed in the plots of average volume temperatures (Figure 6a) in the sample, were also observed when monitoring the average surface temperature on the sample (Figure 6b) during the printing time or the printed height of the sample.

From the liquid fraction plots (Figure 6c), it is even clearer that the gel freezes on the printed sample immediately after leaving the nozzle at the lowest printing speed (500 mm/min). As the printing speed increased, the sample did not cool as quickly, and solidification was minimised, accompanied by an increase in the amount of liquid in the printed sample. At the highest printing speed (1000 mm/min), only 30% of the volume of the printed sample was solidified, which can lead to the deterioration of the final shape of the sample, and thus defects in the printed products. The same is true for the average printing speed (750 mm/min), at which the printed sample was solidified to 65% of its volume.

An important factor in the gel printing process is the storage modulus, which determines whether the gelatine can hold its own weight. The storage modulus of the 10% gel solution is in the range of 0.01–10,000 Pa [5] for the temperature range of the 10% gel solution from 35 °C to 0 °C. In our simulation case, samples of size 10 × 10 mm and maximum height of 2 mm are printed. If we assume that the sample has a total height of 2 mm, the sample has a storage modulus of maximum 20 Pa on the bottom layer of the sample. If the cooling platform is efficient and removes sufficient heat from the extruder section (into which the fresh gel solution is filled at a temperature of 38 °C), the temperature of the gel should not rise above the limiting temperature, which is between 15 °C and 20 °C [5]. According to the published data for a 10% gel solution, this is the point (gel temperature between 15 °C and 20 °C) where the storage modulus is 20 Pa. From the simulations performed at different printing speeds, it appears that at a very high printing speed of 1000 mm/min, the collapse rate of the printed sample increases as a function of the printing speed. At this printing speed, we would reach the maximum sample temperature value of 15 °C already at a sample height of 1.4 mm, which could already be very close to the general conservative limit of 20 Pa storage modulus at a given temperature. This is a conservative criterion that also includes a safety factor, because at this height, the lower part of the sample is already solidified to such an extent that the storage modulus is lower than 20 Pa, which corresponds to the storage modulus of the entire sample height.

Simulation tools were used to confirm that the cryoplatform is capable of handling the increased heat transfer due to variations in printing speed relative to the temperature of the bioproducts up to a certain printing speed. In combination with the well-known storage module [5], the stability of the printed sample can also be predicted based on the simulated temperature profiles, so that critical process parameters for efficient product quality can be avoided in advance of planning the process conditions.

## 3. Conclusions

We developed a simulation model for the cryoplatform by using CFD to simulate the gelatine solidification process during 3D printing. Using a simplified CFD model based on experimental data at given freezing parameters for gelatine solidification, a numerical model was developed that can be used further in various gelatine solidification applications. After validating the numerical model and calibrating the gelatine material properties with the experimental results for a simple gelatine solidification, we simulated the gelatine solidification in the printing application for different printing speeds and evaluated the results against the expectations. As can be seen from the simulation results, the speed of the extruder has a large effect on the solidification, because the cold plate is not able to remove all the heat generated at the top of the sample with a new layer of the warm gelatine solution from the extruder at the given temperature conditions. The failure of printing due to errors in mechanical stability will be minimised if, for the given printing parameters and the shape of the printed samples, such a limiting printing speed can be established before printing using numerical simulation.

## 4. Materials and Methods

### 4.1. Preparation of the Gelatine Solution

Alkaline gelatine type B (from bovine skin, Bloom Strength of ~225, isoelectric point of ~5, Mw of 40–50 kDa) was procured from Sigma Aldrich (Saint Louis, MO, USA). The gelatine was prepared as a 10 wt% (Gel-10%) solution with the help of a hot plate and magnetic stirrer for 2–3 h at a temperature of ~>40 °C.

### 4.2. Thermophysical Characterisations of the Gelatine Solution

The material properties of a gelatine solution needed to describe the physical situation are the thermal conductivity λ, specific heat $c_{p}$, and the density ρ. One must notice that these properties are dependent on the local composition of a material. Fourier’s thermal conductivity equation (Equation (3)), as stated below, defines the interrelationship between the four basic thermophysical properties: λ(T), c(T), ρ(T):

(3) $$\frac{\partial T\left(x,t\right)}{\partial t}=\left[\frac{\lambda \;\left(T\right)}{\rho \left(T\right)\cdot c_{p}\;\left(T\right)\;}\right]\times \Delta T\left(x,t\right)=a\left(T\right)\times \Delta T\left(x,t\right)\;$$

and the thermal diffusivity a(T):

(4) $$\frac{\partial T\left(x,t\right)}{\partial t}=\;a\left(T\right)\times \Delta T\left(x,t\right)\;$$

Due to the uncertainty of the results of any measurement typically derived from the measurement device, the examining model, and the measured samples themselves, an Equipment-Specific Uncertainty (ESU), a Model-Specific Uncertainty (MSU), and a Sample-Related Uncertainty (SSU) are considered as contributors to the combined standard uncertainty of the measurement results. Finally, the uncertainty of the output estimate $U_{c}\left(y\right)$ is calculated and multiplied with a coverage factor *k* = 2, and is represented by Equation (5).

(5) $$u^{2}{}_{c;95\%}\left(y\right)=2\times \sum_{i=1}^{N}{\left(\frac{\partial f}{\partial x_{i}}\right)}^{2}\times u^{2}\left(x_{i}\right)\;$$

#### 4.2.1. DSC Analysis

DSC thermal analysis was carried out for a gelatine solution using a NETZSCH DSC 204F1 Phoenix (Netzsch Gerätebau Gmbh, Graz, Austria) instrument. The experiments were conducted under a He atmosphere with a gas flow of 50 mL/min. The analyses were conducted from 38 °C to −40 °C with a controlled cooling rate of 20 K/min. The DSC measurement curve provides the heat flow (mW) at each temperature for a specific sample mass, and these values are converted to specific heat, $c_{p}$, (J/(g·K)) as a function of temperature.

(6) $$c_{p}{}^{\left(s\right)}\;(T)=\frac{DSC^{\left(s\right)}\left(T\right)-DSC^{\left(B\right)}\left(T\right)}{DSC^{\left(R\right)}\left(T\right)-DSC^{\left(B\right)}\left(T\right)}\times \frac{m^{\left(R\right)}}{m^{\left(s\right)}}\times c_{p}{}^{\left(R\right)}\;(T)$$

The uncertainty of the DSC result of measurement typically derives from the measurement device, the examining model, and the measured samples themselves. To calculate the uncertainty of $c_{p}\left(T\right)$, Equation (5) is applied to Equation (6).

(7) $$u^{2}{}_{c\;}\left(c_{p}\right)=\sum_{i=1}^{N}{\left(\frac{\partial f}{\partial x_{i}}\right)}^{2}\times u^{2}\left(x_{i}\right)\;$$

Here, the ESU|$c_{p}$ is estimated from the deviations in the baselines of the device. Contributions to the MSU|$c_{p}$ result from the uncertainty of the reported $c_{p}\left(T\right)$, data of the reference material used (sapphire), and the contributions to the SSU|$c_{p}$ result from the behaviour of the samples during the measurements. Thus, ESU|$c_{p}$, MSU|$c_{p}$, and SSU|$c_{p}$ are considered as contributors to the combined standard uncertainty of the measurement results, as shown in Equation (7).

#### 4.2.2. Thermal Diffusivity Measurements

The thermal diffusivity measurements of the Gel-10% solution were performed by the Laser Flash Method using a NETZSCH LFA 427 LFA 467 Hyper flash (Netzsch Gerätebau Gmbh, Graz, Austria) with a temperature range of 38 °C to −40 °C under helium conditions to ensure a sufficient thermal link between the sample and the furnace. The thermal diffusivity, a(T), is extracted from the experimental data by the following Equation (8).

(8) $$a\left(T\right)=-\frac{\ln\left(\frac{1}{4}\right)}{\pi }\times \frac{h^{2}\left(T\right)}{t_{\frac{1}{2}}}$$

where a half time *t*_1/2_ is defined as the time when half the maximum temperature increase of 0.5.Δ*T*∞ = Δ*T*(*t*_1/2_) occurs, and h is the thickness of the sample.

In terms of the uncertainty of thermal diffusivity, the application of Equations (2)–(6) only estimates the ESU|a of the flash setup. The MSU|a and the statistical spread of a set of samples must be considered to calculate the standard uncertainty of a flash result, additionally to the ESU|a. So, the uncertainty in terms of thermal diffusivity can be written as follows.

(9) $$u^{2}{}_{c\;}\left(a\right)=\sum_{i=1}^{N}{\left(\frac{\partial f}{\partial x_{i}}\right)}^{2}\times u^{2}\left(x_{i}\right)+u^{2}\left(C_{s}\right)+u^{2}\left(C_{M}\right)\;$$

#### 4.2.3. Thermal Density and Viscosity Analysis

The thermal density and viscosity of the Gel-10% solution at a temperature range of 38 °C to −40 °C was measured by a Stabinger Viscosimeter SVM 3001 (Anton Paar, Graz, Austria) operated under standard atmosphere, but flushed with 5.0 nitrogen. Here, analysis of the U-tube oscillation was used to determine the density of the measured liquid.

### 4.3. Cooling Experiments with the Gelatine Solution

The cryoplatform module set-up was fabricated with stacked Peltier elements (TEC1-12710 with dimensions of 40 × 40 × 3.3 mm) and a copper water cooling block with a dimension of 40 × 40 mm as a heat sink. An aluminium (Al)-based cold plate was used as the surface layer. The cryoplatform plate was cooled down to −20 °C by using a DC power source (0–30 V DC, Neuhold Electronik, Graz, Austria). The gelatine solutions, with a temperature of ~>38 °C, were poured into the Petri dish made of aluminium on the top of a precooled Al-based cold plate. The temperature of the water chamber was kept constant at ~4 ± 1 °C for the entire measuring time. The cooling kinetics of the gelatine solution were recorded by six thermal elements powered by EBI40.

### 4.4. CFD Simulation

#### 4.4.1. Governing Equations and Numerical Methods

The governing equations describe the fundamental physical laws of fluid flow. The equation for the conservation of mass (continuity equation) is written as follows:

(10) $$\frac{\partial \rho }{\partial t}+\nabla \left(\rho \vec{u}\right)=0$$

The balance of momentum is described by:

(11) $$\frac{\partial }{\partial t}\left(\rho \vec{u}\right)+\nabla \left(\rho \vec{u}\vec{u}\right)=-\nabla p+\nabla \left(\overset{=}{\tau }\right)+\rho \vec{g}$$

where $\rho \vec{g}$ represents the gravitational force. The stress tensor $\overset{=}{\tau }$ is given by:

(12) $$\;\overset{=}{\tau }=\mu \left[\left(\nabla \vec{u}+\nabla {\vec{u}}^{T}\right)-\frac{2}{3}\nabla \times \vec{u}I\right]$$

where *I* is the unit tensor. The ANSYS Fluent code solves the energy equation in the following form:

(13) $$\frac{\partial }{\partial t}\left(\rho E\right)+\nabla \left(\vec{u}\left(\rho E+p\right)\right)=\nabla \left(k_{eff}\nabla T-\underset{j}{\sum }h_{j}\vec{J_{j}}+\left({\overset{\approx }{\tau }}_{eff}\cdot \vec{u}\right)\right)+S_{h}$$

where $k_{eff}$ is the effective conductivity (*k* + *kt*, where the second term is the turbulent thermal conductivity) and $(\vec{\;J_{j}}$) is the diffusion flux of species j. The first term on the right-hand side of the equation represents energy transfer due to conduction, the second represents species diffusion, and the third represents viscous dissipation. The term $S_{h}$ includes the heat generated from chemical reactions or any other volumetric heat sources.

#### 4.4.2. Solidification Model

The time-dependent solidification of gelatine was modelled in ANSYS Fluent using the enthalpy porosity technique. In this technique, the melting interface is not tracked explicitly. Instead, a liquid fraction is assigned to each cell in the computational domain, indicating that the fraction of the cell volume that is in liquid form.

The liquid–solid mushy zone is a region where the values of the liquid fraction are between 0 and 1, and where the temperature is between the liquidus ($T_{l}$) and solidus ($T_{s}$) temperatures. The mushy zone is modelled as a “pseudo-porous” medium in which the porosity decreases from 1 to 0 as the material solidifies. When the material solidifies completely, the porosity becomes 0, and thus, the velocities decrease to 0. The enthalpy of the material is calculated as the sum of the sensible enthalpy (*h*) and the latent heat (Δ*H*):

(14) $$H=h+\Delta H=h_{ref}+\int_{T_{ref}}^{T}c_{p}dT+f_{l}L_{v}$$

where $h_{ref}$ is the reference enthalpy, $T_{ref}$ is the reference temperature, $c_{p}$ is the specific heat at constant pressure, $f_{l}$ is the liquid mass fraction, and $L_{v}$ is the latent heat of fusion. The liquid fraction, $f_{l}$, is defined as:

(15) $$f_{l}=\{\begin{array}{cc} 1 & T>T_{l}\\ \left(T-T_{s}\right)/\left(T_{l}-T_{s}\right) & T_{s}<T<T_{l}\\ 0 & T<T_{s} \end{array}$$

Finally, for the solidification problem, the energy equation reads as:

(16) $$\frac{\partial }{\partial t}\left(\rho H\right)+\nabla \left(\rho \vec{v}H\right)=\nabla \left(k\nabla T\right)$$

where H is the enthalpy (see Equation (14)), ρ is density, and $\vec{v}$ is velocity. The conservation equations of mass and momentum are decoupled from the thermal energy equation. These equations are solved using a segregated solver with the second-order accurate upwind scheme.

#### 4.4.3. Geometry and Mesh

**Figure 7:**
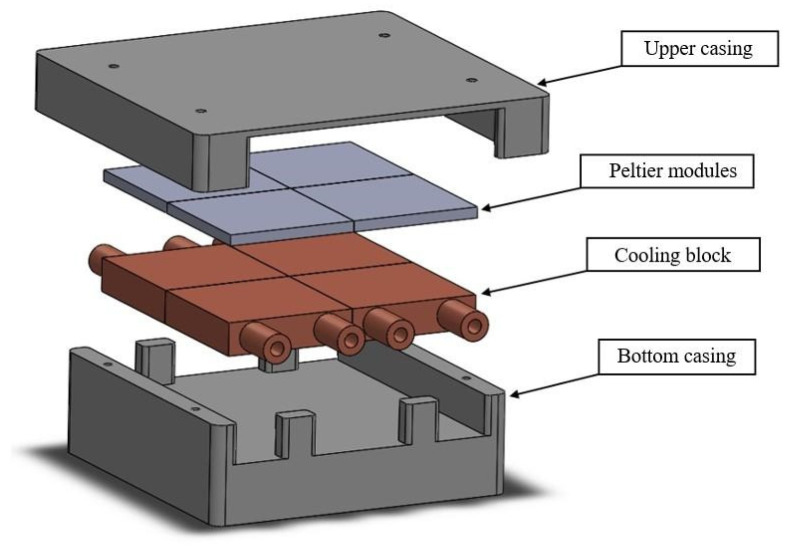
Cooling assembly of bioprinting platform.

The cooling device of the bioprinting platform comprises a lower housing portion, an upper housing portion, four cooling elements, and eight electrical Peltier elements, as shown in Figure 7. The Peltier elements have a colder top and a warmer bottom depending on the current. To lower the temperature of the lower Peltier side, cooling elements made of copper are used, through which the water flow is passed to remove the heat from the system. The simulations were performed in the Ansys software environment. It was necessary to design the basic geometry of the assembly, create a computational grid, and designate the sections that were later used to determine the boundary conditions.

#### 4.4.4. Boundary Conditions

**Figure 8:**
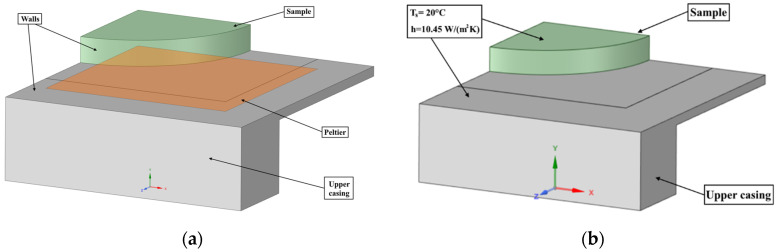
Cooling assembly shown on (**a**) consist of three main elements where the different boundary conditions were prescribed on the surface selections shown on this figure. On figure (**b**) the boundary condition temperature and heat transfer coefficient are given.

On the surface in contact with the Peltier element, named “Peltier” in Figure 8, a constant heat flux of −2000 W/m^2^ was prescribed as a boundary condition (shown in Figure 8). Based on the analytical calculations, the boundary conditions for the heat transfer coefficients were determined from the considered system to the environment. A convective boundary condition with a heat transfer coefficient of 10.45 W/(m^2^K) and room temperature of 20 °C was used for the walls of the upper casing and the sample surfaces in contact with the ambient air. Since the simulations performed were transient simulations, the initial temperature of each body was set. The initial temperature for the aluminium plate was set to −20 °C, and the temperature of the Petri dish containing the biomaterial (located on the plate) was set to 37 °C.

#### 4.4.5. Fluid Properties

Temperature-dependent fluid properties were used. Experimentally measured values were used for the thermal conductivity and specific heat. A constant value of 1000 kg/m^3^ was used due to the small differences in density. Since the biomaterial was mainly water, 0 °C was used for the temperature at which freezing starts, with the heat of fusion of the pure solvent corresponding to 90% water (10% of gelatine). For pure water, the enthalpy of fusion was 333.55 kJ/kg, which corresponded to 300.195 kJ/kg for Gel-10% in our case.

## Figures and Tables

**Table 1:** Temperature-dependent thermal diffusivity (m^2^/s), specific heat capacity (J/g·K), and density (g/cm^3^), plus the calculated thermal conductivity (W/m·K) of the Gel-10% solution with their corresponding measurement uncertainties ($U_{c}$ ).

	Calculated Values		Measured Values					
T	λ	$U_{c}\;\left(\lambda \right)$	*a*	*U_c_* (*a*)	*c_p_*	$U_{c}\left(c_{p}\right)$	ρ	$U_{c}\left(\rho \right)$
*T*/°C	*λ*/W/m·K		*a* × 10^6^/m^2^/s		$c_{p}/J/g\text{·}K$		*ρ*/g/cm^3^	
40	0.5227	0.089984	0.1338	0.016634	3.9012	0.037555	1.0015	0.11886
38	0.5107	0.073104	0.1344	0.018374	3.8035	0.082070	0.9987	0.036643
30	0.5115	0.068069	0.1382	0.010748	3.6897	0.381668	1.0030	0.031100
25	0.5017	0.143145	0.1396	0.009615	3.5070	0.969591	1.0250	0.015800
20	0.4546	0.052582	0.1386	0.010748	3.2011	0.258152	1.0250	0.030000
10	0.3479	0.147735	0.1394	0.010307	2.4340	1.015339	1.0250	0.030000
0	1.2885	0.148143	0.6227	0.021750	2.0189	0.213102	1.0250	0.030000
−10	1.5512	0.065615	0.7800	0.011077	1.9403	0.052454	1.0250	0.030000
−20	1.5784	0.071809	0.7999	0.024982	1.9251	0.029684	1.0250	0.030000
−30	1.5305	0.149932	0.8153	0.075092	1.8315	0.029336	1.0250	0.030000
−40	1.4766	0.817094	0.8230	0.454528	1.7504	0.031749	1.0250	0.030000

**Table 2:** Printing speeds for different simulation variants.

	P1	P2	P3
Printing speed (mm/min)	500	750	1000

## Data Availability

The data presented in this study are available on request from the corresponding author.
